# Effect of redroot pigweed interference on antioxidant enzyme and light response of common bean (*Phaseolus vulgaris* L.) depends on cultivars and growth stages

**DOI:** 10.1038/s41598-023-31466-2

**Published:** 2023-03-15

**Authors:** Seyede Zahra Tabatabaiepour, Zahra Tahmasebi, Alireza Taab, Sajad Rashidi-Monfared

**Affiliations:** 1 Department of Agronomy and Plant Breeding, Faculty of Agricultural, Ilam University, Ilam, Iran; 2 Agricultural Biotechnology Department, Faculty of Agriculture, Tarbiat Modares University, Tehran, Iran

**Keywords:** Biotechnology, Ecology, Molecular biology

## Abstract

Redroot Pigweed (*Amaranthus retroflexus* L.) is an important weed that is highly competitive with common bean. Photosynthetic pigments, the activity of antioxidant enzymes, the relative expression of a number of antioxidant enzyme and light response genes, were studied in three of common bean cultivars and in V4 and R7 stages under Redroot Pigweed free and infested. The presence of weeds reduced the content of chlorophyll, relative chlorophyll and anthocyanin of common bean leaves. With the increase of weed competition, the expression of antioxidant genes and enzymes increased, which indicates the increase of their activity in order to reduce the amount of reactive oxygen species. Among the studied antioxidant enzymes, the activity of catalase and ascorbate peroxidase produced in the leaves was higher than that of superoxide dismutase. With the increase of weed interference, the expression of *phytochrome interacting factor 3* (*PIF3*) gene as a positive regulator of light signals is increased and the expression of *phytochrome rapidly regulated1* (*PAR1*) gene as a negative regulator is decreased. *Chlorophyll a/b-binding protein* (*CAB1*) and *auxin-responsive protein IAA8* (*IAA8*) genes also down-regulated with increasing competition. Along with the decrease of *CAB* expression in the conditions of competition with weeds, the chlorophyll a, b content also decreased. Correlation between gene expression and physiological traits related to them highlights the prominent role of CWCP in maintaining yield potential.

## Introduction

Common bean (*Phaseolus vulgaris* L.) as an annual summer crop is one of the most important crops in the legume family. The common bean cultivation was estimated to be 33,066,183 hectares in 2019^1^. It is also an important source of protein (16 to 37%), carbohydrates and calories^2,3^. Therefore, common beans play an important role in human nutrition^3^.

Weed interference is one of the main biological constraints in common bean production^4,5^. Weed interference reduces the dry grain yield of common beans by up to 85%.

As a result of weed interference, a 31–55% decrease in grain yield was obtained in black bean genotypes, and a decrease in weed growth was seen in genotypes with higher yields^6^. Early growth of volunteer corn and competition with beans caused a decrease in leaf area, height and dry matter biomass of bean cultivars^7^. Black bean competition with alexandergrass for resources, in addition to reducing the productivity of bean crop, has a negative effect on a some of morphological variables such as plant height, number of trifoliate leaves, number of pods per plant, number of grains per pod, dry mass and mass of one thousand grains^8^. Late growth of volunteer corn resulted in lower yield losses (11.1 to 19.7%) of IPP Gralha and IPR Uirapuru bean cultivars compared to early growth of weeds at the same time as bean emergence (19.6 to 35.5%). Early weed growth before bean growth or at the same time as bean growth increases the competition and eventually increases the yield loss^9^. The most sensitive growth stage of the bean plant to weed interference is the vegetative growth stage^10^. Rapid early growth of the crop allows more light and nutrients to be absorbed than fast-growing weeds; The initial growth of the root is also important for water and food competition, and the initial growth of the stem is also related to the growth of the root; Even during plant establishment, root growth and competition for nutrient and water uptake may be more important than competition for light^11^. Kembel and Cahill^12^ found that the contact of the roots with a different environment compared to the shoot, causes a different correlation of above and below ground traits in response to environmental variables. A meta-analysis on the studies relating roots and shoots during competition under nutrient stress showed that roots were subjected to much more competition than stems; So that during this intense competition, biomass decreased by 42%^13^.

Weed allelopathic activities early in the season may limit the crop root system development and growth^2^.

The critical period of weed control has been reported to be from 3 to 5 or 6 weeks after planting in common beans^14^ and from the second trifoliate stage to early flowering in white common bean^15^. There are several predominant weeds commonly found in the common bean fields.

The high competitive ability of the Redroot Pigweed (*Amaranthus retroflexus* L.) against crops is due to important morphological and physiological characteristics such as high height, C4 photosynthetic pathway, deep and transverse root expansion, abundant seed production, high light extinction coefficient and high growth rate^16^.

The photosynthetic capacity of plants depends on abiotic factors such as the quality and quantity of light. Each of the photosynthetic pigments such as chlorophyll a, b and carotenoids absorb light at a different wavelengths^4^. The rate of photosynthesis and biomass production in plants is also largely dependent on the chlorophyll content of leaves^17^. The weed interference in the growth of soybean plant caused a decrease in the content of carotenoids and chlorophyll pigments in soybean leaves^18^.

Dense vegetation causes a change in the quality of received light, and plants perceive the presence of nearby plants through changes in light quality. Before shading occurs, the ratio of red (R) to far-red (FR) light decreases due to the reflection of FR by the plant tissue; Low R:FR is perceived by phytochrome photoreceptors and induces a shade avoidance response^19^; One of the characteristics of the shade avoidance response is accelerated elongation growth of leaf-bearing organs. Low R:FR inactivates phytochromes and causes accumulation and activation of transcription factors PHYTOCHROME-INTERACTING FACTORs (PIFs) 4, 5, and 7 and subsequent expression of their growth-mediating targets. During true shading, especially in the red (R) and blue (B) wavelengths, the amount of transmitted light is reduced due to absorption by chlorophyll^20^.

The study of changes in the production of reactive oxygen species (ROS) in crops in response to weed interference, has demonstrated changes in enzymatic activity and gene expression of antioxidant compounds in relation to oxidative stress. Stress signaling due to weed interference in crops^21^ might be related to light conditions^22^ and allelopathic compounds released from weeds. Far red light reflected from adjacent weeds induces changes in the expression profiles of scavenger genes of ROS^23^. The high rate of far-red light results in the production of ROS^24^ that affects both photosynthesis and carbon partitioning^25^. Allelochemicals stimulation of ROS production and activation of antioxidant-mediated defense may lead to damage to DNA, proteins, and cell membranes^26,27^. Competition of Italian ryegrass (*Lolium multiflorum*) has led to oxidative damage to and increased activity of SOD, CAT, and APX enzymes in soybean^28^. Therefore, it is important to quantify the dynamic expression of antioxidant genes, as well as changes in the activity of enzymes and compounds that serve as tools to combat ROS that finally affect the crop yield^27,29^.

Genes in crop plants are irreversibly altered by the presence of weeds^23,30^. Weeds also cause crop developmental changes by reducing the regulation of genes necessary for nutrient uptake by roots^31^. Up and down and down-regulation of defense-related genes in plants has been reported^32–36^. Down expression of photosynthetic genes due to interspecific competition has been reported in various studies^30,37,38^. *Phytochrome B* (*phyB*) gene was upregulated in both barley and maize at high plant density^39^. PIFs (phytochrome interacting factor) inhibits carotenoid accumulation by down-regulating the expression of the Phytoene Synthase (*PSY*) encoding gene, which is the major enzyme that determines velocity in the carotenoid pathway^40^. A study indicated roles for increased oxidative stress and jasmonic acid signaling responses during weed stress^35^. According to transcriptome studies shade avoidance was found to be necessary as part of soybean response to weeds during the critical weed control period (CWCP)^35^, while shade avoidance responses in similar transcriptome studies has not been shown^37^.

There is no information on changes in gene expression that occurred in common bean under weeds interference during the CWCP period. Therefore, the aims of this study were to evaluate the expression of a number of antioxidant enzyme and light response genes and investigate the activity of antioxidant enzymes, anthocyanins and photosynthetic pigments of several red common bean cultivars in third trifoliate leaf (V4) and pod formation (R7) growth stages under weed free and weedy conditions during the CWCP period.

## Results

### Gene expression of antioxidant enzymes

**Figure 1:**
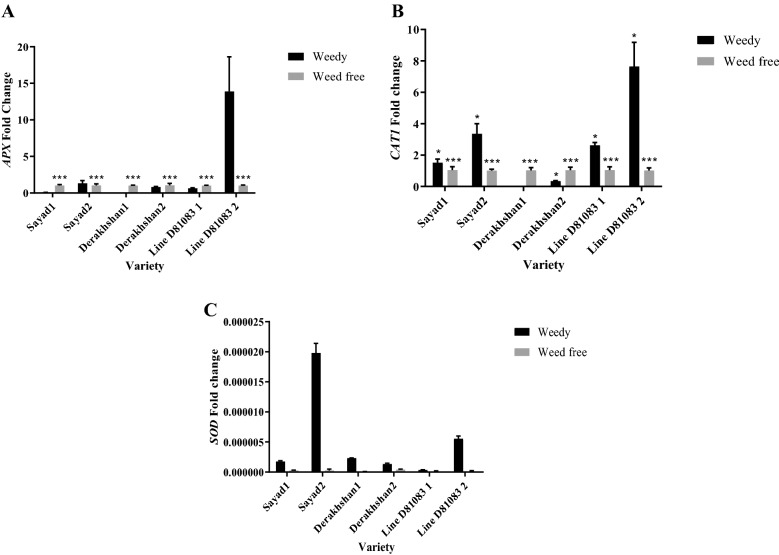
Real-time PCR (qRT-PCR) analysis of transcription levels of *APX* (**A**), *CAT1* (**B**) and *SOD* (**C**) genes in two treatments: weedy and weed free, in the three cultivars/lines studied in two stages of sampling V4 and R7 (data are the mean ± standard error in five biological replications and five technical replications, numbers 1 and 2 next to the name of the cultivar represent the V4 and R7 stages, respectively). ***: *P* < 0.99; *: *P* < 0.90.

The results showed an increase in *APX*, *CAT1,* and *SOD* expression in common bean cultivars/lines except for the *SOD* gene in the Derakhshan cultivar (Fig. 1A,B,C) in stage R7 compared to stage V4 upon competing with weeds. Upon weed interference and in the R7 stage, the level of *CAT1* gene transcript was increased 7.64 and 3.35 folds in lines D81083 and Sayad cultivar, respectively (Fig. 1B).

### Expression of genes related to light and photosynthesis

**Figure 2:**
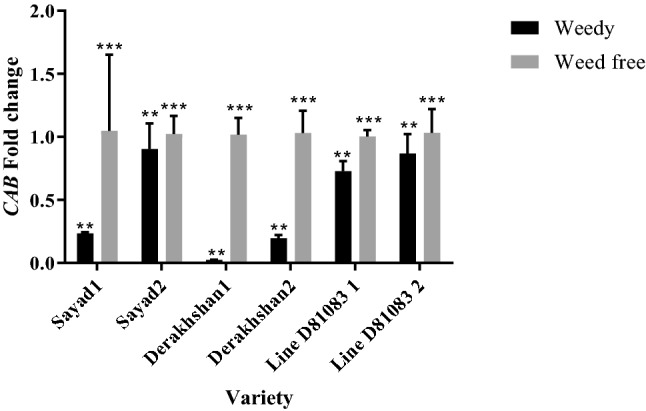
Real-time PCR (qRT-PCR) analysis of *CAB* gene transcript levels in two treatments weedy and weed free, in the three cultivars/lines studied and in two stages of sampling V4 and R7 (data are the mean ± standard error in five biological replications and five technical replications, numbers 1 and 2 next to the name of the cultivar represent the V4 and R7 stages, respectively). ***: *P* < 0.99; **: *P* < 0.95.

Decreased expression of the *CAB* gene (chlorophyll a/b-binding protein) was observed in both V4 and R7 stages. The highest expression of this gene was observed in the V4 stage in line D81083 (0.72 times) while its lowest expression was detected in the Derakhshan cultivar (0.024 times). The highest and lowest *CAB* gene expression was reported in the R7 stage in Sayad and Derakhshan cultivars (showing an increase by 0.90 and 0.19) respectively (Fig. 2).

**Figure 3:**
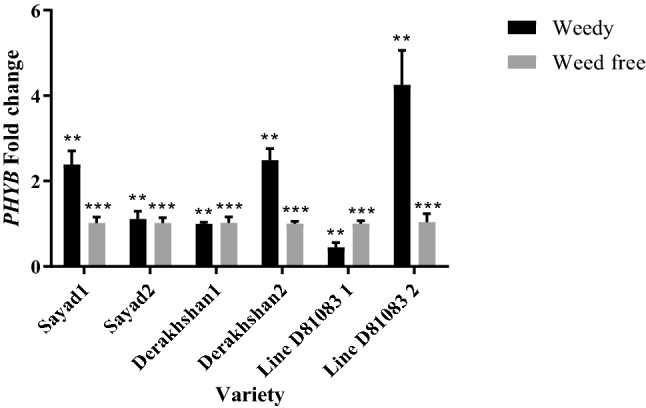
Real-time PCR (qRT-PCR) analysis of *PHYB *gene transcript levels in two treatments weedy and weed free, in the three cultivars/lines studied and in two stages of sampling V4 and R7 (data are the mean ± standard error in five biological replications and five technical replications, numbers 1 and 2 next to the name of the cultivar represent the V4 and R7 stages, respectively). ***: *P* < 0.99; **: *P* < 0.95.

Evaluation of *PHYB* (*Phytochrome B*) gene expression in weedy and weed-free conditions in the V4 stage (Fig. 3) showed that the expression of this gene was higher under complete weed interference in Sayad cultivar (2.38 times) as compared with the weed-controlled state. It was, however, lower in Derakhshan cultivar and line D81083. The highest and lowest expression levels of the *PHYB* gene at the R7 stage were 4.25 and 1.11 times, respectively, in D81083 and Sayad lines.

**Figure 4:**
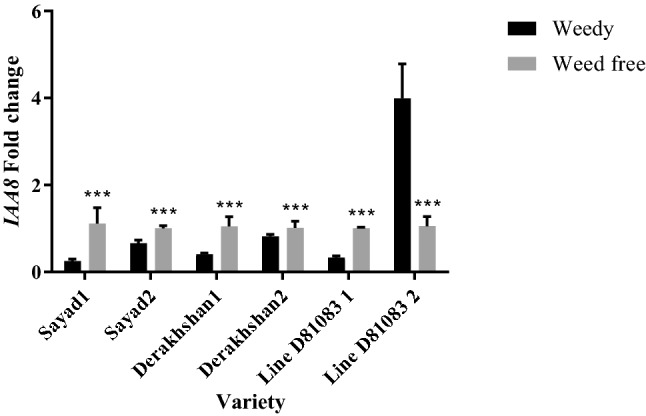
Real-time PCR (qRT-PCR) analysis of *IAA8 *gene transcript levels in two treatments weedy and weed free, in the three cultivars/lines studied and in two stages of sampling V4 and R7 (data are the mean ± standard error in five biological replications and five technical replications, numbers 1 and 2 next to the name of the cultivar represent the V4 and R7 stages, respectively). ***: *P* < 0.99.

Under complete weed interference, decreased expression of the *IAA8* (auxin-responsive protein) gene was observed in the V4 stage of common bean growth in the three cultivars/lines studied (Fig. 4). The highest and lowest expression of this gene at the V4 stage were 0.40 and 0.24 times in Derakhshan and Sayad cultivars, respectively. The highest and lowest expression of the *IAA8* gene at the R7 stage were observed in line D81083 (3.98 times) and Sayad (0.65 times), respectively.

**Figure 5:**
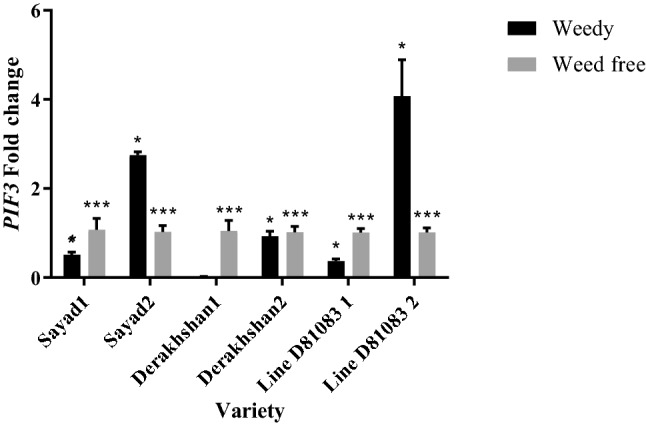
Real-time PCR (qRT-PCR) analysis of *PIF3 *gene transcript levels in two treatments weedy and weed free, in the three cultivars/lines studied and in two stages of sampling V4 and R7 (data are the mean ± standard error in five biological replications and five technical replications, numbers 1 and 2 next to the name of the cultivar represent the V4 and R7 stages, respectively). ***: *P* < 0.99; **: *P* < 0.90.

Decreased expression of the *PIF3* gene (phytochrome 3 interacting factor) was observed at the V4 stage under complete weed interference in the three studied cultivars/lines (Fig. 5). The highest and lowest expression of the *PIF3* gene at the V4 stage were observed in Sayad cultivar (0.51 times) and Derakhshan cultivar (0.019 times), respectively. At the R7 stage, an increase was detected in the expression of this gene in Sayad and D81083 cultivars under complete weed interference, while showing a slight decrease in Derakhshan cultivar. The highest and lowest expression of the *PIF3* gene at the R7 stage were observed in line D81083 (4.072 times) and Derakhshan cultivar (0.92 times), respectively.

**Figure 6:**
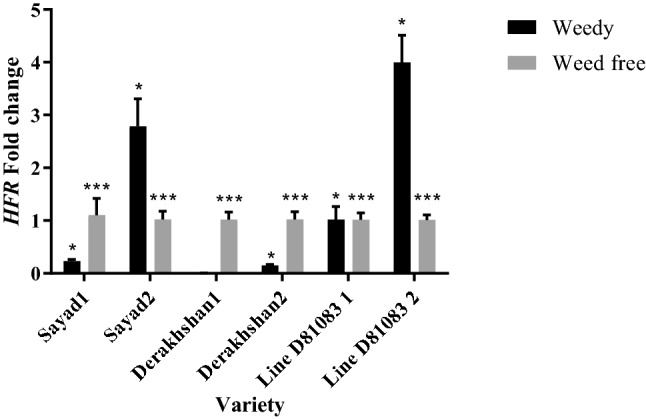
Real-time PCR (qRT-PCR) analysis of *HFR *gene transcript levels in two treatments weedy and weed free, in the three cultivars/lines studied and in two stages of sampling V4 and R7 (data are the mean ± standard error in five biological replications and five technical replications, numbers 1 and 2 next to the name of the cultivar represent the V4 and R7 stages, respectively). ***: *P* < 0.99; **: *P* < 0.90.

Results of *HFR* gene expression (Long Hypocotyl in Far-Red light) under complete weed interference at the V4 stage (Fig. 6) showed that the expression of this gene decreased in Sayad (0.23 times) and Derakhshan (0.003 times) cultivars while exhibiting a slight increase in D81083 line (1.016 times). At the R7 stage, expression of this gene increased with incrementing weed interference pressure on the common bean plant. Increased *HFR* gene expression was also observed in Sayad cultivar and line D81083 under complete weed interference; whereas a decrease was observed in its expression in the Derakhshan cultivar. The highest and lowest expression of the *HFR* gene at the R7 stage were reported in the D81083 line (3.99 times) and Derakhshan cultivar (0.146 times), respectively.

**Figure 7:**
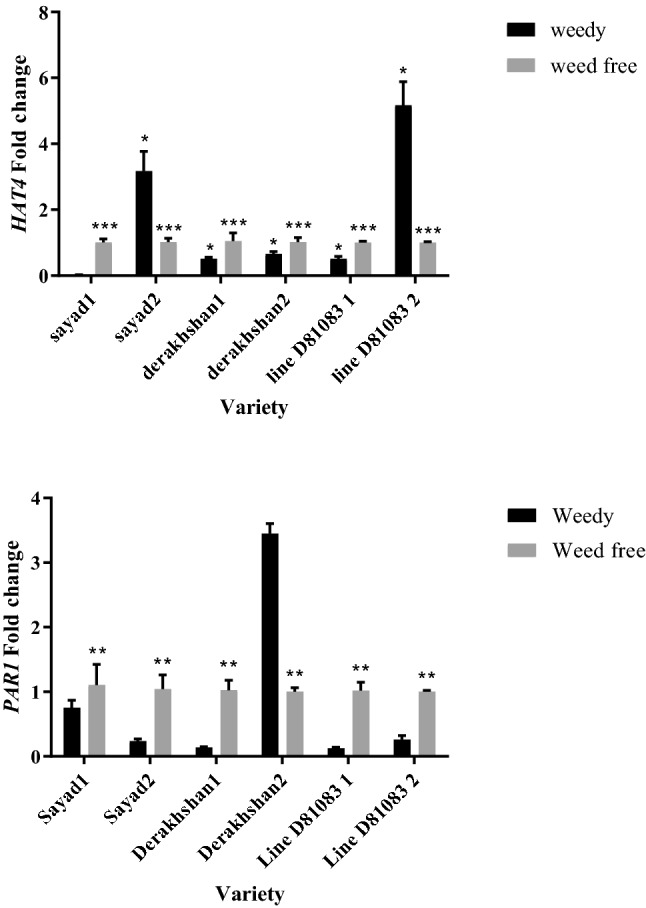
Real-time PCR (qRT-PCR) analysis of *HAT4 and PAR1* genes transcript levels in two treatments weedy and weed free, in the three cultivars/lines studied and in two stages of sampling V4 and R7 (data are the mean ± standard error in five biological replications and five technical replications, numbers 1 and 2 next to the name of the cultivar represent the V4 and R7 stages, respectively). ***: *P* < 0.99; **: *P* < 0.95; *: *P* < 0.90.

Decreased expression of the *HAT4* gene (Homeobox-leucine zipper protein HAT4) was detected under complete interference with weeds (weedy) at the V4 stage of common bean growth in all three studied cultivars/lines (Fig. 7). The highest *HAT4* expression at the V4 stage was observed in the Derakhshan cultivar (0.509 times) while its lowest expression level was recorded in the Sayad cultivar (0.018 times). At the R7 stage, the expression of this gene increased with prolonging the weed interference duration. A significant rise was detected in the expression of this gene in Sayad cultivar and line D81083 exposed to complete weed interference increased but decreased while the Derakhshan cultivar showed a decline. The highest and lowest expression of the *HAT4* gene at the R7 stage were 5.167 and 0.653, respectively, observed in line D81083 and Derakhshan cultivar.

Investigation of the expression of the *PAR1* gene (rapid regulation of phytochrome) under complete weed interference at the V4 stage indicated decreased expression in the three studied cultivars/lines (Fig. 7). The highest and lowest expression of the *PAR1* gene at the V4 stage was observed in the Sayad cultivar (0.75 times) and the D81083 line (0.12 times), respectively. The expression level of this gene increased at the R7 stage by prolonging the weed interference period in the Derakhshan cultivar and D81083 line; while showing a decline in the Sayad cultivar. Decreased *PAR1* gene expression was observed under complete weed interference in Sayad cultivar (0.23 times) and D81083 line (0.25 times) whereas Derakhshan cultivar exhibited an increase (3.44 times).

### Physiological traits

#### Antioxidant enzymes

**Figure 8:**
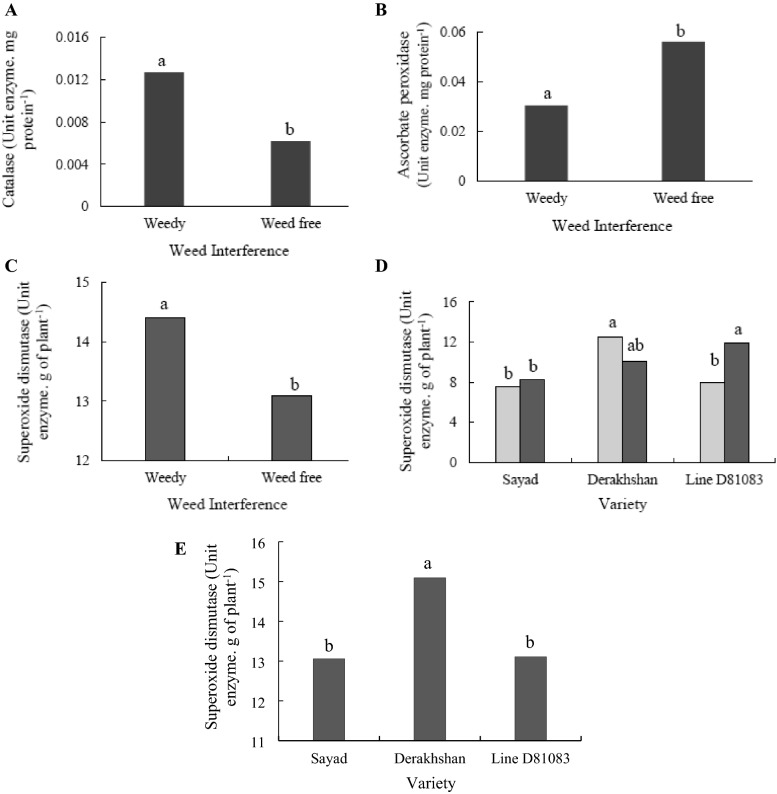
The activity of antioxidant enzymes: (**A**) Catalase content in the V4 stage in two treatments weedy and weed free; (**B**) Ascorbate peroxidas content in V4 stage in two treatments weedy and weed free; (**C**) Super oxide dismutase content in R7 stage in two treatments weedy and weed free; (**D**) Super oxide dismutase content among the cultivars studied in two treatments weedy and weed free in V4 stage (Gray column: weedy; Black column: weed free); (**E**) Super oxide dismutase content among the cultivars studied in Rv stage.

Weed interference treatment with 0.0127 enzyme units per mg of protein led to the highest level of catalase in the V4 stage (Fig. 8A). The catalase and ascorbate peroxidase contents of the R7 stage were not affected by any of the treatments. At the V4 stage and under weed interference, the ascorbate peroxidase content (0.0599 units of enzyme per mg of protein) decreased compared to weed control conditions (0.03047 units of enzyme per mg of protein) (Fig. 8B).

At full interference with weeds and the V4 stage, the Derakhshan cultivar showed the highest superoxide dismutase content (12.525 enzyme units per gram of plant material) while the Sayad cultivar demonstrated the lowest amount of this enzyme (7.5608 enzymatic units per gram of plant material) (Fig. 8D). At the R7 stage, based on Fig. 8C, the content of superoxide dismutase enzyme was higher under the complete weed interference (14.41 units of enzyme per gram of plant material) as compared to the weed-free state (13,025 units of enzyme per gram of plant material). At the R7 stage, the cultivars Derakhshan and Sayad had the highest and lowest amount of superoxide dismutase enzyme, respectively (Fig. 8E).

#### Chlorophyll

**Figure 9:**
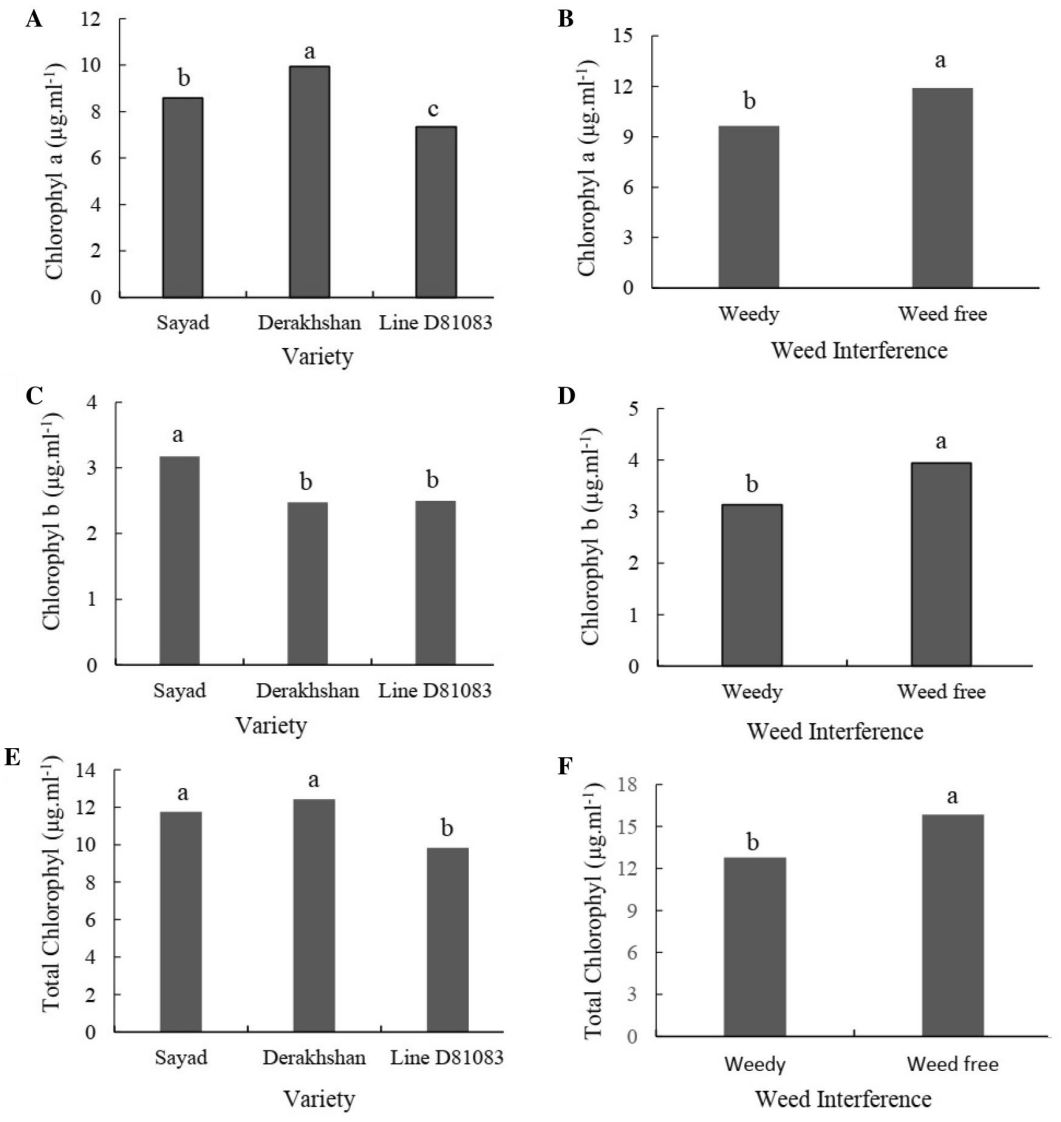
The chlorophyll-a (**A** and **B**), chlorophyll-b (**C** and **D**) and total chlorophyll content (**E** and **F**) in two treatments weedy and weed free, in the three cultivars/lines studied and in two stages of sampling V4 and R7.

**Figure 10:**
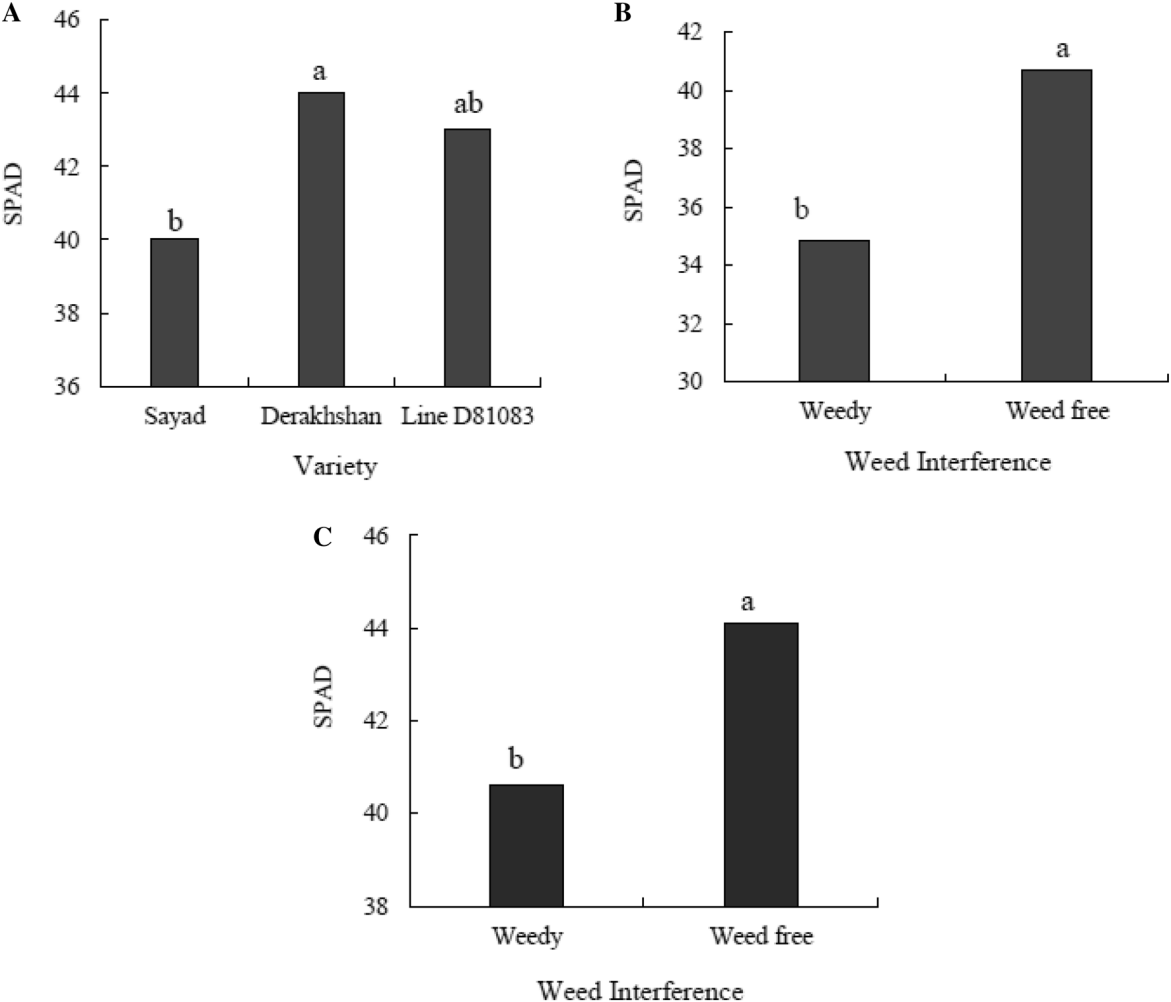
Relative chlorophyll content in the three cultivars/lines (**A**), two treatments weedy and weed free (**B**) and in two stages of sampling V4 and R7 (**C**).

At the V4 stage, Derakhshan cultivar and line D81083 had the highest (9.94 μg/ml) and the lowest (7.33 μg/ml) chlorophyll a (Fig. 9A), respectively. As shown in Fig. 9B, at the R7 stage, the chlorophyll-a content was lower under complete interference with weeds as compared to the weed-controlled conditions. At stage R7, the amount of chlorophyll-a respectively increased by 16.36% and 24.22% in the plants exposed to complete weed interference and weed-free control conditions as compared to the V4 stage. At V4 stage, the Sayad and Derakhshan cultivars had the highest (3.17 μg/ml) and lowest (2.47 μg/ml) chlorophyll-b contents, respectively (Fig. 9C). As shown in Fig. 9D, at the R7 stage, the chlorophyll-b content was lower under complete weed interference (3.13 μg/ml) compared to the weed-free state (3.94 μg/ml). Liters). In stage V4, Derakhshan cultivar and line D81083 had the highest (12.42 μg/ml) and lowest (9.84 μg/ml) total chlorophyll content (Fig. 9E), respectively. In both V4 and R7 stages, the total chlorophyll content was higher under completely controlled weed conditions as compared with the weed interference state. The total chlorophyll content respectively increased by 15.80 and 24.81% at the R7 stage under complete weed interference and the weed-free state as compared to the V4 stage (Fig. 9F). The highest and lowest relative chlorophyll levels at the R7 stage were observed in Derakhshan and Sayad cultivars, respectively. Relative chlorophyll content increased at the R7 stage compared to the V4 stage. Compared to the V4 stage, the relative chlorophyll content respectively increased by 14.30 and 7.66% in the R7 stage for samples exposed to complete weed interference and complete weed control (Fig. 10).

#### Realtion between chlorophyll a, b and content some expression genes in both V4 and R7 stages

Decreased expression of the genes related to light and photosynthesis was observed in V4 stage in compared to the weed-free treatment; the chlorophyll a and b content also in this treatment are decrease and have a direct relationship with each other. The results of the chlorophyll a and b content (Fig. 9) and the expression of *CAB*, *IAA* and *PAR1* genes in the R7 stage show that at simultaneously with the down-regulation of this genes in the weedy treatment, the chlorophyll a and b content also in this treatment are decrease and have a direct relationship with each other. Exposure to low R:FR from the weedy treatment in the R7 stage resulted in up-regulation of *PHYB*, *PIF3*, *HFR* and *HAT4* genes which have an inverse relationship with the chlorophyll a and b content; of course, exceptions were seen in some cultivars in response to weedy treatment. In the V4 stage, in the weedy treatment, compared to the weed free treatment, there is an inverse relationship between the decrease in the chlorophyll a and b content and up-regulation of *CAT* and *SOD* genes, and a direct relationship with down-regulation of the *APX* gene. In the R7 stage, there is an inverse relationship between the decrease in the chlorophyll a and b content and up-regulation of *CAT*, *SOD* and *APX* genes in the weedy treatment.

#### Anthocyanins

**Figure 11:**
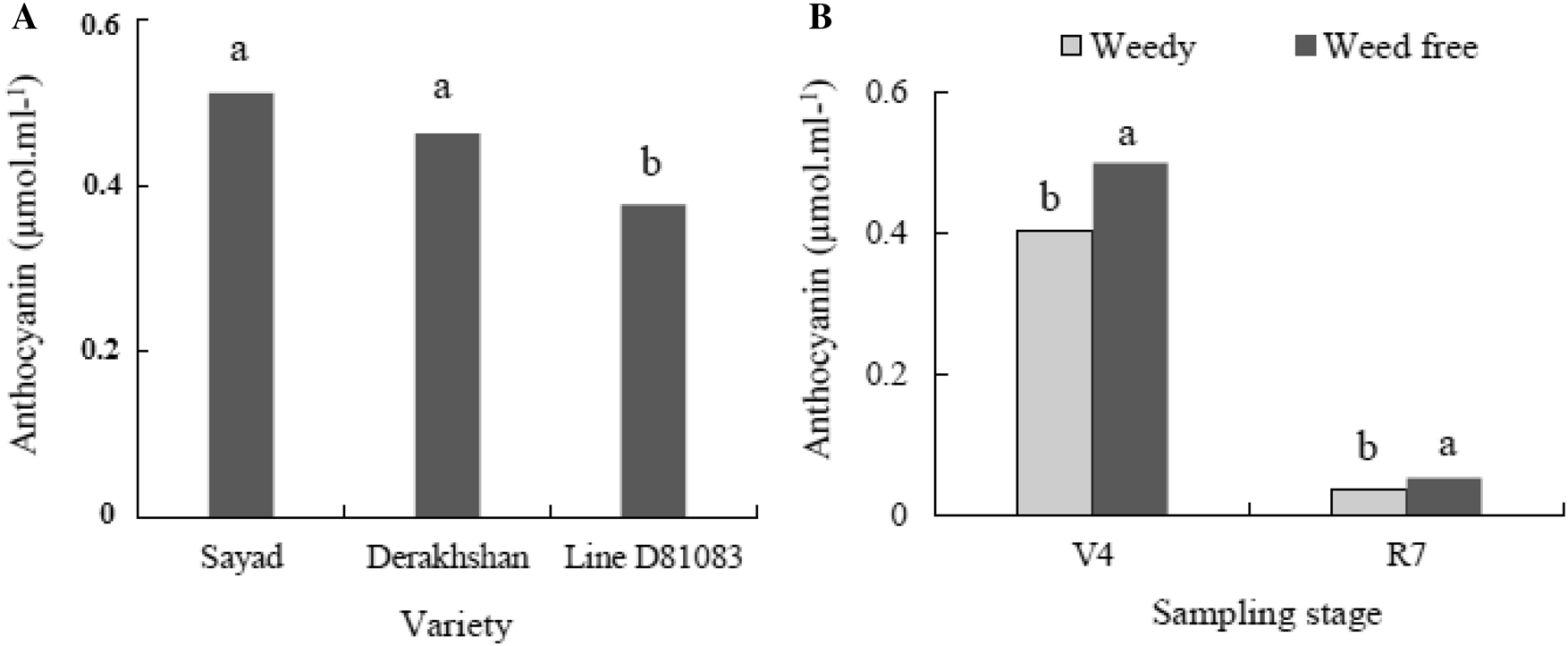
The anthocyanin content in the three cultivars/lines (**A**) and two treatments weedy and weed free and in two stages of sampling V4 and R7 (**B**).

The highest and lowest anthocyanin levels at the V4 stage were observed in the Sayad cultivar (0.511 μmol/ml) and line D81083 (0.3758), respectively (Fig. 11A). Anthocyanin levels decreased at the R7 stage compared to the V4 stage (approximately 90%) (Fig. 11B).

#### Carotenoids

**Figure 12:**
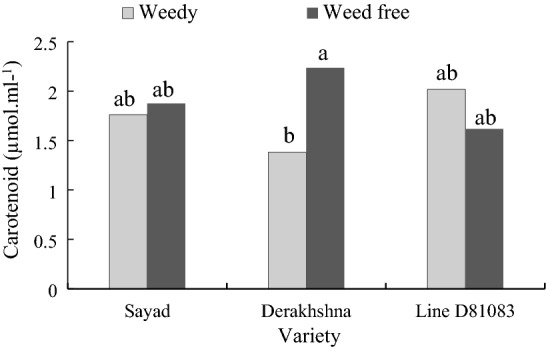
The carotenoid content in two treatments weedy and weed free, in the three cultivars/lines studied and in two stages of sampling V4 and R7.

At the V4 stage, the Derakhshan cultivar had the highest carotenoids content (2.2350 μmol/ml) under complete weed control, while line D81083 exhibited the highest amount of carotenoids (2.091 μmol/mL) under weed interference (Fig. 12).

## Discussion

In both stages, an increase was observed in *CAT1* gene expression of Sayad cultivar and line D81083 that was pronounced at R7 under weed conditions. However, no gene expression was observed in the V4 stage with slight expression in the R7 stage in the Derakhshan cultivar under weed interference (Fig. 1B). At the R7 stage, the content of Antioxidant enzymes under the complete weed interference was higher as compared to the weed-free state.Increased and decreased expression of ROS scavenging genes were observed in the hypocotyl stage and the single leaflet stage of soybean development, respectively^23^. The production of ROS under different types of stress is a common mechanism. Imbalance in the production and removal of ROS due to environmental stresses can lead to a rapid and unstable ROS rise and oxidative damage to the plants, resulting in the activation of defense mechanisms against oxidative stress and, hence, increased activity of antioxidant enzymes in plant cells^22,28,41^. The presence of weeds at densities greater than or equal to crop can damage the cell membrane by lipid peroxidation and leakage of cell contents into the environment^42,43^ suggesting weed control measures before the effects emerge. The R/FR ratio of light reflected from adjacent plants and transmitted to the crop can alter the amount of leaf antioxidants. Shade-compatible responses control the accumulation of antioxidants in leaves through the R/FR signal transmission system. The reflected R/FR ratio can act as a warning signal, informing the plants about the increased risk of photon inhibition and oxidative stress^44^. Under FR-rich conditions, changes in the content of photosystem elements (PS) lead to the production of ROS as well as antioxidant responses. According to a study by Mckenzie-Gopsill et al.^18^, weed-filled treatment significantly increased H_2_O_2_ levels in monocotyledonous leaves (19%) by increasing the activity of waste-removing enzymes. ROS was associated with ascorbate peroxidase (30%) and glutathione peroxidase (14%) but catalase activity remained unchanged^18^.

The expression of *CAB* gene (chlorophyll a/b-binding protein) decreased at the R4 and R7 stage with enhancing the weed interference pressure on the common bean plant. A small number of selected genes were expressed in maize under weed stress at the V8 stage that included different members of the CAB family; a senescence-related protein, and the glycosyltransferase 8 family^37^. Decreased expression of genes involved in photosynthesis (such as ribulose bisphosphate carboxylase and chlorophyll a-b binding protein) was observed in corn under competition with velvetleaf^45^. Low expression of this gene confirms that weed stress and shade limit the photosynthetic abilities of the plant. In both V4 and R7 stages, the total chlorophyll content was higher under completely controlled weed conditions as compared with the weed interference state. FR-rich light generated by adjacent weeds affected the levels of chlorophyll precursors (such as Pchlide and Chlide a)^46,47^. However, the effect of increasing FR light on photosynthetic pigments varies between plant species and in-plant organs^46^.

Under complete weed interference, prolonged weed competition enhanced the expression of *Phytochrome B* at the R7 stage in the Derakhshan cultivar (line D81083) while it showed a decline in the Sayad cultivar. R/FR light signals are received and transmitted by phytochrome light receivers. Arabidopsis contains several phytochrome receptors (*PHYA-E*^48^). These different receptors have somewhat common roles in shade avoidance syndrome (SAS), day and night regulation, seed germination, and seasonal growth changes such as bud dormancy and flowering^35^. Masclaux et al.^32^ identified the *PHYA* gene which is involved in the transmission of light signals. *PIF3* gene expression was constantly increased in response to the presence of weeds^35^.

Under complete weed interference, with increasing the duration of weed interference, the expression of the *IAA8* gene increased at the R7 stage compared to the V4 stage, however, the gene expression decreased compared to the complete weed-controlled Sayad and Derakhshan cultivars while showing a rise in D81083 line. In the study of maize microarrays in competition with velvetleaf^37^, *Aux/IAA* gene expression decreased. Previous findings suggest that both the auxin and ethylene signal transduction responses are altered by shade avoidance responses^49,50^.

The expression of *PIF3* gene increased at the R7 stage with enhancing the weed competition pressure on the common bean plant. PIF3 is a helix-loop-helix transcription factor^51^ that is more regulated by post-transcriptional mechanisms^52^. In a study on soybean RNAseq in competition with weeds, the weed-induced *PIF3* gene (*PIF3a*) was clearly increased at the presence of weeds, but if weeds were removed in the V3 stage, weed-induced *PIF3a* expression did not maintain its high level^35^.

*HFR* gene expression under complete weed interference at the V4 stage decreased while At the R7 stage, expression of this gene increased. *HFR1* is one of the well-known negative regulators induced by low R/FR treatment^53^. This negative regulator prevents more elongation at a low R/FR ratio^19^. A comparison of Kim et al.^53^ study with previous reports showed no strong association with several marker genes for the shadow avoidance response such as *ATHB2*, *HFR1*, *FT* and other auxin-related genes.

Expression of the *HAT4* gene under complete interference with weeds (weedy) at the V4 stage of common bean decreased but at the R7 stage increased. Masclaux et al.^32^ identified several specific genes which respond to low R/FR ratios, these genes are involved either in light signal transmission (*HFR1*, *PHYA*, *FHL*), or elongation processes regulated by auxin (*HAT2*, *XTR7*), or auxin transport (*ASA1*). This indicates that plants grown at high densities detect changes in light quality and initiate a growth response regulated by auxin.

In addition, expression of the *PAR1* gene under complete weed interference at the V4 stage decreased and at the R7 stage increased. *PAR1* (*Phytochrome rapidly Regulated 1*) and *PAR2* are negative regulator genes that originated from avoiding low R/FR-induced shading in *Arabidopsis*, which may not be induced in response to weed competition^19,35^.

Anthocyanin levels decreased at the R7 stage compared to the V4 stage. In weed-filled treatments, exposure to maize at low R/FR ratios decreased the stem anthocyanins while increasing lignin in stem tissues compared with weed-free treatments^24^. Besseau et al.^54^ reported that the metabolic change in the phenylpropanoid pathway can occur through different pathways to produce anthocyanins or lignin. Anthocyanins are required to protect the plant against high light conditions and will be reduced under weed-induced shade conditions^35^.

Infrared-rich light (FR-E) was also increased under weed conditions. It has been shown to affect carotenoid levels^46,47^. However, the effect of FR-E light on photosynthetic pigments depends on the plant species and in-plant organs^46^. In a study by Mckenzie-Gopsill et al.^18^, the levels of leaf carotenoids, as the strongest ^1^O2 waste remover, were significantly reduced under light conditions in weed-filled treatment (39%).

## Conclusion

By enhancing the pressure of weed interference on the common bean plant, the expression of most studied genes increased at the R7 stage compared to the V4 stage. Sayad cultivar and line D81083 exhibited the highest catalase contents under complete weed interference whereas these varieties demonstrated the lowest amount of catalase enzyme under weed-free conditions and at the V4 stage. Despite no significant differences in ascorbate peroxidase of different treatments, the activity of this enzyme showed a 67% increase under competition with weeds during the R7 stage as compared to the V4 stage. In both stages, the total chlorophyll content was higher under weed-controlled conditions compared to the full interference with weeds. Under weed interference, line D81083 had the highest amount of carotenoids. It can be generally concluded that adjacent weeds alter the proportion of photosynthetic pigments, and induce specific hormonal and defense responses. Weeds negatively affect photosynthesis and growth. Changes in gene expression negatively influence the growth of the plant. Under competition with weeds, the common bean induces the expression of genes involved in light quality signal transmission processes (low R/FR). Moreover, weeds continuously alter the growth responses and gene expression in the common bean plant. The gene expression of the common bean plant alters with the developmental stage; the antioxidant system of the common bean plant behaves differently under weed stress. Adjacent weeds increase the level of leaf ROS and affect ROS scavenger network components. The highest increase in gene expression and enzymatic activity was observed at the R7 stage (pod formation). Thus, it can be concluded that the weed interaction with the common bean plant reached its highest level as the R7 stage. This study showed that crop-weed interactions are much more complex than previously thought. It can be concluded response of common bean to competition with weed depends on its growth stage and genotype. The study of transcriptomes and gene expression responses in common bean plants under competition with weeds along with physiological and morphological studies provides an opportunity to deeply explore key issues in weed science.

The relationship of studied genes to genes involved in shade avoidance responses in *Arabidopsis* and other listed plants, provide evidence that these genes may be important in the response of common been to weeds. These results suggest that the studied genes can will be a target for manipulating weed tolerance in common been. Various researchers have suggested manipulating the shade avoidance response as a means to improve weed tolerance in crops^35,55^. Such experiments could provide much information to improve the competitive ability of crop genotypes. In addition, this information is needed to develop of crop–weed interactions to better understand and predict the consequences of weed competition and interference. Future examination should provide us with a better model to gain new insights into the mechanism underlying the regulation of antioxidative defense system in common bean in the Redroot Pigweed Interference responses.

The timing of weed emergence and duration pf weed competition have an important effect on crop yield, and studies have shown that just a few days of early growth by the crop relative to weeds can significantly shift the competitive balance in favor of the crop^56^. With the early planting of common bean plant when the temperature is lower, the beginning of the growth of the crop plant is faced with the growth and less competition with weeds and can help the growth of beans.

Understanding the molecular mechanisms that mediate the effects of photoreceptor signals and regulate the expression of plant genes, allows the researcher to manipulate planting density and canopy structure to optimize light penetration and to improve plant performance. Also, this understanding of functional information can help create crop cultivars that maintain elevated levels of defense even at high planting densities^57^.

Breeding of crops in the direction canopy development and greater leaf area during the critical weed control period (CWCP) (V4 to R7) by using the methods like screening and biotechnology is another promising strategy for efficient weed control.

The limitations on photosynthesis and physiological responses may be attributed to biochemical limitation on the Calvin cycle. These limitations are preventable if common bean is maintained weed-free during vegetative growth stages throughout the CPWC. Correlation between gene expression and physiological traits related to them highlights the prominent role of CWCP in maintaining yield potential.

## Methods

### Plant materials and experimental setup

A factorial experiment was conducted based on completely randomized design with two factors and five replications at laboratory of Tarbiat Modares University in 2019 the red common bean cultivars/line were obtained from the Agricultural and Natural Resources Research Center of Markazi Province—Khomein National Common Bean Research Station, Iran (Table 1) were set as first factor and weedy and weed free conditions as second factor levels.

**Table 1:** Common bean cultivars used in the experiment.

Cultivars	Origin	Average height (cm)	Growth period (days)	Growing habit
Sayad	Colombia	60–55	90	Unlimited and semi erect growth (Type 2)
Derakhshan	Colombia	40–35	95–100	Limited and erect growth (Type 1)
D81083	Colombia	35	80	Limited and erect growth (Type 1)

The pots with diameter of 25 cm were filled with a mixture of soil, sand, gravel, peat moss and perlite in a ratio of 1: 1: 1: 1: 3, respectively. The soil mixture was autoclaved before filling the pots. Redroot pigweed seeds were stored 6 months at room temperature to reduce their dormancy^58^. A naturally occurring weed population of redroot pigweed that was collected from a red common bean field in 2018 and used as the weed competition source. Redroot pigweed seed were sown in tray before common bean planting to provide seedlings and kept in the greenhouse until the four true leaves were emerged^59^. One common bean seed was planted in the center of each pot at a depth of 4 to 5 cm from the soil surface and 4 redroot pigweed seedlings were transplanted around it^35^. Redroot pigweed plants were grown at a distance of 12 cm from the common bean plant. The height of the Redroot pigweed plant had reached 15 to 20 cm in the stage V4 beans, and it had reached 45 to 50 cm in the stage R7 of the bean plant. The plants were grown in the growth chamber under photoperiod of 16 h' light and 8 h' darkness at a temperature of 25 ± 1 °C, close to the range of optimum temperature for germination and growth of *A. retroflexus*^60^. Light quality (photosynthetic photon flux density, PPFD) was measured using a FS-3080 intelligent photosynthetic and was ≈ 400 μmol m^−2^ s^−1^ for the duration of the experiments. Irrigation was done once every four days by adding a constant amount of water to each pot.

### Plant sampling

Sampling of common bean plants were done at V4 (third trifoliate leaf stage) as beginning weed interference and at R7 stage (pod formation) in all treatments. For both treatments, sampling was done at the same time of the day (9–10 am). Two leaf samples were selected from the top three leaves in each pot and sealed in sterile aluminum foil and immediately frozen in liquid nitrogen and then transferred to a − 80 °C freezer for RNA extraction and study the physiological traits.

Less than half of the leaf lamina of the common bean is under the shadow of the weed; of course, the whole bean plant (root, leaf and stem) is involved in competition with weeds. In the Rv stage, the shading of the weed was more on the common bean leaves. The test conditions have not changed, but the amount of interference between common bean and weeds and the shading of weeds on the bean plant increases.

### Gene expression analysis by qRT-PCR

Extracting the total RNA was performed by powdering 0.1 g (100 mg) of frozen common bean leaf tissue in liquid nitrogen, using Qiagen kit (RNeasy Plant Mini Kit), according to manufacturer's instructions (Qiagen, www.qiagen.com). After RNA extraction, the quantity (concentration) of RNA was measured using Epoch microplate spectrophotometer (BioTek Company, USA). The quality of RNA samples was determined by electrophoresis of the samples on 1% agarose gel in TAE buffer. Synthesis of the first strand of cDNA from total RNA was performed using protocol of the company Fermentas (www.thermofisher.com).

To analyze gene expression, ten genes of genes involved in crop and weed interference were selected (Table 2). Genes whose expression in common bean plants were examined in two V4 and R7 stages, were selected based on previous studies on the competition of different plant species with weeds^19,23,32,34,35,53,61^.

**Table 2:** Primers characteristics used in the study.

Full gene names	Primer name	Accession number	Forward primer (F) (5' → 3')	Reverse primer (F) (5' → 3')	Product length (bp)	T_m_ (°C) Forward primer	T_m_ (°C) Reverse primer
*Phytochrome B*	*PHYB*	KF775132.1	CCTTTTCTGGTTCAGGTCGC	CCATTTCCGCATTCTCCCAT	167	56.3	55.5
*chlorophyll a/b-binding protein*	*CAB*	JX869947.1	ATGTTCGGGTTCTTCGTCCA	AACTCTCGAATCCACAAGTCATTC	162	56.4	55
*opper/zinc superoxide dismutase*	*SOD*	KF569535.1	GCTGTTGTTGTCCATGCTGA	CGCCCGTTTTCATGTGACTA	139	56	55.4
*ascorbate peroxidase*	*APX*	KF033563.1	CCTTCTTCGCTGATTACGCA	GGCAAACACACCCTCACATC	131	55.2	56.6
*catalase1*	*CAT1*	AF149283.1	GGCACATGGATGGTTTTGGT	GTGGCATGACTGTGGTTGAA	157	56.2	55.7
*auxin-responsive protein IAA8*	*IAA8*	KF033420.1	GGGATGTACCGTGGGAAATG	GATGAGACAAAAGGCAGTCCC	144	55.7	55.9
*phytochrome interacting factor 3*	*PIF3*	XM_007163190.1	GCAGAATCCAGTGTCCTTTCAT	ACCAAATTCTACAGTCGCCTCC	163	55.2	57
*Long Hypocotyl in Far-Red Light*	*HFR*	XM_007144565.1	CGAACGGGAAGAAGGTTAGC	ATACACATTAAGCCATTGTGATTGG	169	55.7	55.7
*homeobox-leucine zipper protein HAT4*	*HAT4*	KF569529.1	CCGCCACACGTCATCATC	TTCACACCCTTCCCTCCA	139	56.4	55.8
*Phytochrome Rapidly Regulated*	*PAR1*	–	CCTTGCAGTACCAGGTGAAAG	GCTGCTGCTTCCATAATCCATC	185	55.8	56.3
*Actin11*	*ACT11*	62703083	TGCATACGTTGGTGATGAGG	AGCCTTGGGGTTAAGAGGAG	190	55	56.4

In order to design the primers of the studied genes, their protected sequences (selection of the forward primer from the coding sequence (5'CDS) and the reverse primer from the 3'UTR (Untranslated Regions) of the gene from the NCBI site with the address http://www.ncbi.nlm.nih.gov was taken. Then, the primers related to these sequences were used using Primer3 software with the web address http://frodo.wi.mit.edu/primer3, Primer—Blast and Oligo 7 (Version: 7.54) were designed and the final evaluation and approval of the primers was performed using the online Oligoanalyzer software (https://www.idtdna.com/pages/tools/oligoanalyzer) and for primer synthesis was sent to the Metabion company in Germany. The *ACT11* reference gene was also used to correct and determine the relative expression of the target genes. This gene is usually involved in essential cell processes^62^.

Quantitative analysis of gene expression was performed using a QIAGEN Rotor-Gene Q (5plex HRM Platform) real-time device. For quantitative evaluation of gene expression, CTs related to the main genes and internal control genes (five technical replicates and three biological replicates) obtained from real-time PCR, using Rest software and based on Levak equation (Eq. 1)^63^ were analyzed. GraphPad Prism software was also used to plot gene expression data.

1 $$\begin{gathered} \Delta {\text{Ct}} = C_{t}^{Sample } - C_{t}^{control} \hfill \\ {\text{R}} = 2^{{ - \left[ {\Delta Ct Sample - \Delta Ct Control } \right]}} \hfill \\ \end{gathered}$$

### Measurement of anthocyanin and photosynthetic pigments content

To measure the amount of chlorophylls a (Eq. 2) and b (Eq. 3), total chlorophyll (Eq. 4)^64^, anthocyanin (Eq. 5)^65^ and carotenoid (Eq. 6)^66^, 200 mg of each selected green leaf were homogenized in 5 ml of 80% acetone. After centrifugation the samples at 3000 rpm and temperature of 4 °C for 15 min, the supernatant was removed and the volume was increased to 10 ml with 80% acetone. Then, the absorption rate was measured at 663, 470, 646.6, 663, 647 and 537 wavelengths by UV–Visible spectrophotometer (Cary-50 model made by Varian company, Australia), and photosynthetic pigments were calculated using the following equations. A sample of 80% acetone was used as a control to adjust the device.

2 $${\text{Chl}}.{\text{a }}\left( {\upmu {\text{g}}\,{\text{ml}}^{{ - {1}}} } \right) = {12}.{25}\left( {{\text{A 663}}.{6}} \right){-}{2}.{55}\left( {{\text{A 646}}.{6}} \right)$$

3 $${\text{Chl}}.{\text{b}}\left( {\upmu {\text{g}}\,{\text{ml}}^{{ - {1}}} } \right) = {2}0.{31}\left( {{\text{A 646}}.{6}} \right){-}{ 4}.{91}\left( {{\text{A 663}}.{6}} \right)$$

4 $${\text{Chl}}.{\text{T}}\left( {\upmu {\text{g}}\,{\text{ml}}^{{ - {1}}} } \right) = {17}.{76}\left( {{\text{A 646}}.{6}} \right) + {7}.{34}\left( {{\text{A 663}}.{6}} \right) = {\text{Chl}}.{\text{a}} + {\text{Chl}}.{\text{b}}$$

5 $${\text{Anthocyanin}}\left( {\upmu {\text{mol ml}}^{{ - {1}}} } \right) = 0.0{8173}\left( {\text{A 537}} \right){-}0.00{697}\left( {\text{A 647}} \right){-}0.00{2228}\left( {\text{A 663}} \right)$$

6 $${\text{Carotenoid}}\left ( {\upmu {\text{g}}\,{\text{ml}}^{{ - {1}}} } \right) = \left[ {{1}000\left( {{\text{A 47}}0} \right){-}{3}.{27}\left( {{\text{Chl}}.{\text{a}}} \right){-}{1}0{4}\left( {{\text{Chl}}.{\text{b}}} \right)} \right]/{229}$$

where A is the rate of absorption of the extract solution at specified wavelengths. Chl.a, Chl.b and Chl.T are the concentrations of chlorophyll a, chlorophyll b and total chlorophyll, respectively. Carotenoids include carotene and xanthophyll.

### Enzymatic activity analysis

For enzymatic extract, 200 mg of common bean leaf sample were prepared. The activity of catalase enzyme was measured at 25 ± 1 °C using a spectrophotometer. Catalase activity was measured by calculating the reduction of H_2_O_2_ uptake at 240 nm. Enzyme activity was calculated in the form of the enzymatic unit according to the total protein (mg) obtained by Bradford (67) method in 100 μl of enzyme extract per minute. A unit of enzymatic activity is considered as the amount of enzyme that breaks down 1 mmol of H_2_O_2_ in one minute^67^.

One enzymatic unit of ascorbate peroxidase is the amount of enzyme that oxidizes one millimole of ascorbate per minute. Using absorption changes at 290 nm, ascorbate extinction coefficient (2.8 mMol^−1^ cm^−1^) and formula A = εbc, the amount of ascorbate remaining was calculated after 2 min of enzymatic reaction. Over time, the rate of absorption has a decreasing trend. Thus an enzyme unit of ascorbate peroxidase is the amount of enzyme that oxidizes one millimole of ascorbate in one minute. Enzyme activity according to enzyme units was reported in the amount of total protein (mg) obtained by Bradford method^68^ in 100 μl of enzyme extract^69^.

Superoxide dismutase (SOD) activity was determined by measuring the photochemical reduction inhibition of nitroblue tetrazolium (NBT) at 560 nm^70^. Sample adsorption divided by blank absorption shows the percentage of inhibition as SOD activity. One unit of SOD activity is defined as the amount of enzyme required to inhibit the photochemical reduction of NBT by nearly 50%. The amount of SOD activity was obtained as the unit of enzyme per gram of plant material. All procedures were conducted in accordance with the guidelines.

### Statistical analysis

The normality and homogeneity of variances in data were assessed using SPSS 22 software. Analysis of variance for the physiological data was performed by a factorial design based on a completely randomized design using SAS 9.4 software. The mean comparison s of the studied traits was performed using the least significant difference (LSD) test in SAS 9.4 software at the significant level of 5%.

## Data Availability

All data analyzed during this study are included in this article. The required genes were extracted from the NCBI database (see https://www.ncbi.nlm.nih.gov/) in order to design primers and then study gene expression. Accession numbers of studied genes are available in Table 2. The genetic resources are available (in the Agricultural and Natural Resources Research Center of Markazi Province—Khomeini National Common Bean Research Station, Iran).
